# Programmatic Access to FAIRified Digital Plant Genetic Resources

**DOI:** 10.1515/jib-2019-0060

**Published:** 2020-01-08

**Authors:** Mehmood Ghaffar, Danuta Schüler, Patrick König, Daniel Arend, Astrid Junker, Uwe Scholz, Matthias Lange

**Affiliations:** Leibniz Institute of Plant Genetics and Crop Plant Research (IPK) Gatersleben, Corrensstrasse 3, 06466 Seeland, Germany

**Keywords:** plant digital resources, genotyping, phenotyping, lab information management, FAIR data

## Abstract

Genetic variance within the genotype of population and its mapping to phenotype variance in a systematic and high throughput manner is of interest for biodiversity and breeding research. Beside the established and efficient high throughput genotype technologies, phenotype capabilities got increased focus in the last decade. This results in an increasing amount of phenotype data from well scaling, automated sensor platform. Thus, data stewardship is a central component to make experimental data from multiple domains interoperable and re-usable. To ensure a standard and comprehensive sharing of scientific and experimental data among domain experts, FAIR data principles are utilized for machine read-ability and scale-ability. In this context, BrAPI consortium, provides a comprehensive and commonly agreed FAIRed guidelines to offer a BrAPI layered scientific data in a RESTful manner. This paper presents the concepts, best practices and implementations to meet these challenges. As one of the worlds leading plant research institutes it is of vital interest for the IPK-Gatersleben to transform legacy data infrastructures into a bio-digital resource center for plant genetics resources (PGR). This paper also demonstrates the benefits of integrated database back-ends, established data stewardship processes, and FAIR data exposition in a machine-readable, highly scalable programmatic interfaces.

## 1 Introduction

Plant phenomics is an interdisciplinary research domain involving biology, physics, data science, and biotechnology to study plant phenotypes systematically [1]. This must encompass detailed documentation of investigation, observation and measurement of structural as well as functional plant traits in their environmental context. This encompasses observations, quantitative phenotyping and molecular phenotyping [2]. Innovative solutions cover for a range of applications including field, greenhouse and laboratory using various selection of sensors [3]. Furthermore, the systematic reprocessing and digitization of historic phenotypic data as well as environmental data is now become an important task in plant breeding applications.

### 1.1 High throughput plant phenotyping

As an international leading center of crop research, the Leibniz Institute of Plant Genetics and Crop Plants Research (IPK-Gatersleben) feels obligated to this challenge. Numerous plant phenotyping facilities are operating, such as automated greenhouses plant screening [4], which produce an enormous amount of phenotypic data that gets increasing attention as scientific asset in the form of cited data publication and data downloads, e.g. growth screening that has been frequently downloaded (https://doi.ipk-gatersleben.de/report) [5]. High-throughput phenotyping facilities for small, mid-size and large plants under controlled conditions has been installed in the last decade. These systems are capable of assessing traits of a plant from seedling to their pre-mature state in a non-destructive, precise and high throughput manner.

Climate controlled greenhouses are capable to accommodate several thousand plant pots in parallel. Each plant pot possess a unique identifier attached to its base and is transported through special computer controlled carriers. At watering and weighing station, each plant is precisely watered and nourished. The system is also equipped with three different chambers to measure phenotyping of individual plants. In the first chamber, various lighting and camera sensors records water estimation and analysis of photosynthetic activity in each plant. Inside the second chamber, the plants are imaged in a visible light spectrum to observe growth related parameter and architectural traits such as height, bio-mass, and projected leaf area. The growth dynamics will be then documented for several months and can be seen in an animated fashion (time-lapsed animation of plant growth). In the third and last chamber, the plant is irradiated by blue light to emit red fluorescent light which give information about plant vitality. More than 200 phenotype features for each plant are captured each day. That sums a large quantity of phenotype data.

### 1.2 Data management back-ends

To efficiently store and manage data, the use of state-of-the-art database management back-end infrastructure is indispensable. In this context well aligned binary data storage, Relational Database Management System (RDBMS), homogeneous designed data schemata, customized data import and user interfaces must be combined into a all-in-one back-end. Laboratory Information Management Systems (LIMS) are commonly used as anchor component to integrate these components. At IPK, it is decided to use commercial systems to guarantee a long-term system maintenance, and further development of new features and customer support scenario. We found that contracting marked established companies, like ORACLE [6] for the database systems and storage server as well as LIMS-software company [7] AAC-Infotray is efficient model to operate the systems as reliable service but keep resources for scientific work, research and innovation. In contrast, self-developed systems or open source systems bind a notifiable amount of personal resource for service task and have an inherent danger for discontinuity after project end or loss of staff [8].

### 1.3 Data sharing

Besides these back-end services, efficient data-sharing and data-access is a further challenging task for scientific data engineering. Data re-use and exchange is a key component to gain scientific knowledge in natural sciences [9] and particularly in plant science [10]. To reuse these data effectively, an open infrastructure is required that meet the standards as FAIR guidelines (findable, accessible, interoperable, reusable) [11]. To do so, IPK operates the e!DAL-PGP research data publication system [12] and further publish research data records in their native format. In order to implement a FAIR and machine-readable access, the Breeding-API consortium [13] developed a comprehensive set of open RESTful API calls and data formats and definitions to describe a plant phenotype experiment. It mainly focuses on the phenotype experiment and contextual associated marker information. The Global Alliance for Genomics and Health (GA4GH) is another consortium aiming at developing a framework for the documentation and sharing of genomics data. These specifications are set to cope with huge amount of genomes and sequence data [14]. Subsequently we discuss the major challenges to implement the BrAPI and GA4GH specifications for an integrative and harmonize access to all genotypic and phenotypic data in IPK’s institutional databases.

## 2 Concepts and implementation of FAIRified digital PGR

In this contribution, we discuss experiences, concepts and implementations to meet the technical and socio-cultural challenges to operate a FAIR and machine readable data management infrastructure. The challenge of engaging the data producing facilities with backbone infrastructure services is analogous to the “last mile” challenge in telecommunications or transportation. The effort and the complexity of building a FAIR infrastructure for data providers and data users is high when compared to the core infrastructure itself. These efforts vary based on “distance” of the instrument and personal from FAIR services in practice. As consequence, we designed a seamless integration of lab work flows, sensorics instruments and data provisioning into a data processing workflow comprising of a homogeneous LIMS, standard operating procedures, data stewardship, and interoperable and machine-readable data access interfaces.

### 2.1 Central laboratory information management system

In order to implement a sustainable, cost efficient and long-term operational research data management strategy the IPK directors board decided to support the centralisation of scattered data management. Since 2002, a central RDBMS is maintained as long-term service by central Bioinformatics group. This homogeneous platform to host structured data has been used as back-end for the majority of IPK’s information systems and databases (https://www.ipk-gatersleben.de/en/databases/). Despite this technical back-end homogenization, the developed database were designed and implemented in a less interoperable and non-integrative manner. The data structures, interfaces, consistency rules, processing logic and user access control was not compatible to each other. Furthermore, the data maintenance as well as data import, export and graphical user interfaces were also heterogeneous. This resulted in a hardly maintainable systems, especially after expiration of project funding.

**Figure 1:**
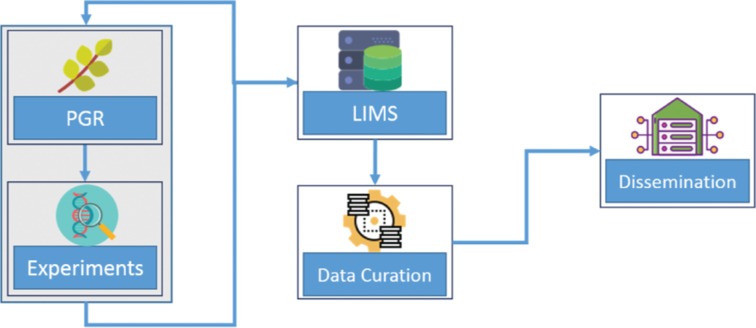
Data processing in LIMS throughout from data-entry to data-dissemination.

**Figure 2:**
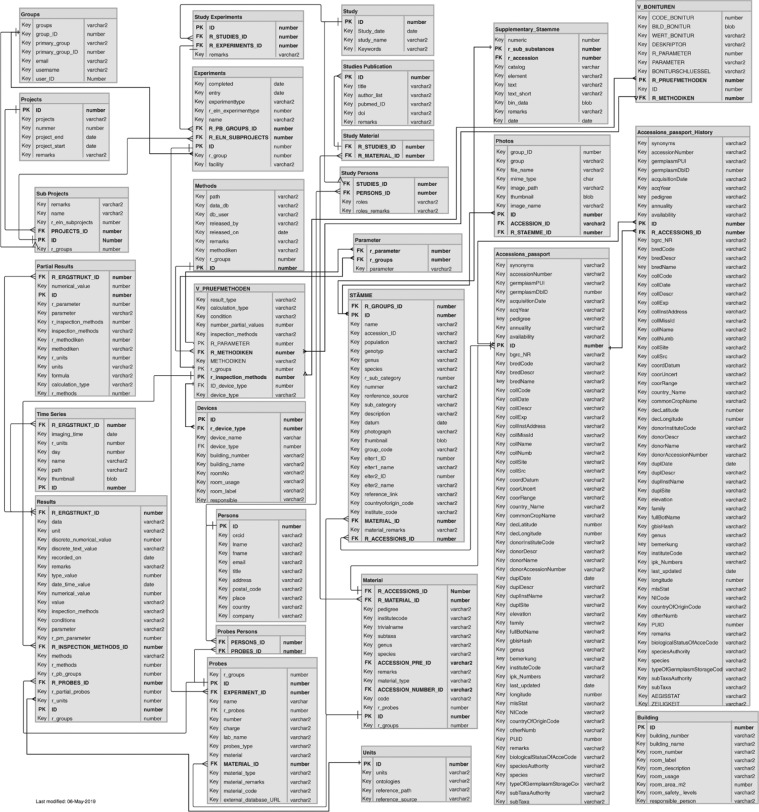
Generic ER schema of the LIMS database backend.

To meet these challenges, the follow-up decision was to implement one homogeneous, multi-purpose database that bundle the so far isolated systems. To implement this, the LIMS “LIMSOPHY” from the company AAC-Infotray was licensed and a permanent staff position was granted to operate this system and became since 2010 a part of institutional strategy for research data management. The general process of LIMS digital enrichment is illustrated in Figure 1. The database back-end is an open relational designed data schema encompassing a flexible user interface and data import features. The data schema models a hierarchical relationship from projects, experiments, probes, materials, parameters and measurement values (see Figure 2). In particular, projects contain all relevant information, i.e. who is responsible for this project, when it started and when it ended, and other information based on ontologies. Projects consist of one or many experiments that are set to find specific research questions. Each experiment uses samples for investigation purposes and produces results. Meta data of used plant material, i.e. accessions originating from IPK *ex situ* gene bank, is synchronized from material donors database systems, like the IPK Gene Bank Information System (GBIS) [15]. Breeding and further plant materials is described by lines and line-information. Any measurement result as well as descriptions of experimental parameters and factors are stored in the result tables. Single measurements can be grouped into time series, such as sequences of images for one sample at different time points or other experimental factors.

### 2.2 Data stewardship

Currently, more than 2.7 million probes and more than 16 million scalar analysis results are stored in the IPK LIMS. Furthermore, around two million binary files are linked. Together these data sets represent IPK primary research data, e.g. sequence data, photographs, and mass spectrometry data. The data is maintained independently in a federal fashion by service labs, scientists and is spread over projects and research groups. Although the common data schema ensures a structural consistency, data stewards are required to keep a reliable data quality and consistency in respect of controlled vocabulary scales and units. Subsequent examples illustrate this issue and are shown in Table 1. Ambiguous values are entered in the labs, such as “first blossom”, “50% of plants”, for the parameter “Inflorescences Slices” and “minimum” and “maximum” for the parameter “Leaf width” and “Flowering Season” respectively. Further problems such as the use of non-homogeneous or incomplete material naming like “Morex_32”, “Morex_42” or “Morex”, which are actually lines of the species Hordeum vulgare subspecies Morex (see Table 2).

**Table 1:** Example of ambiguous attribute values in column “Condition”.

Parameter	Condition	Methods	Result
Inflorescence Slices	First Blossom	Measurement	60
Inflorescence Slices	50% of Plants	Measurement	62
Leaf width	Minimum	Measurement	−2
Leaf width	Maximum	Measurement	−2
Leaf width	Minimum	Measurement	−2
Leaf width	Maximum	Measurement	−2
Flowering Season	Minimum	Measurement	86
Flowering Season	Maximum	Measurement	86

**Table 2:** Non-homogeneous and incomplete material naming.

Material			
TrivialName	Species	Genus	SubTaxa
Morex_32	vulgare	Hordeum	
Morex_42	vulgare	Hordeum	
Morex_52	vulgare	Hordeum	
Morex_11	vulgare	Hordeum	
Morex	vulgare	Hordeum	
A thaliana Sap			

The interpretation of this data requires knowledge of the experimental context, and is depending in practice by individual lab scientist or technician who performed that experiment. In order to make data inter-operable and re-usable for automated, machine-readable data analysis pipelines, this would obviously create a problem for a scientific tool built on top of these output data. In case of data sharing or publication, an explicit and qualified material description is indispensable to make the data effectively re-usable. This, in turn, is an investment that has no direct benefit for the individual scientist as long as the journals and funding agencies does accept paper manuscripts or project reports.

To overcome these issues, data stewardship needs to be embedded as part of project data handling. The success of investing person month from project grants for data stewardship was tested in a pilot project that activated a FAIR access to genotyping and phenotyping data of more than 22.000 barley accessions of the IPK genebank [16] including imaging of hundreds of plants phenotyped in infrastructures of the German plant phenotyping consortium (DPPN; https://dppn.plant-phenotyping-network.de/). With this promising results and because of the scientific and socio-economic value of FAIR data, IPK invest into sustainable strategy within German infrastructure programs for national research data infrastructure (NFDI; https://www.dfg.de/en/research_funding/programmes/nfdi), the German network of Bioinformatics Infrastructure (de.NBI) [17] and ELIXIR [18] to set-up permanent FAIR-data services. These processes support the development of biological collections to bio-digital resources centers [19].

### 2.3 BrAPI – breeding application programming interface

The Minimal Information About Plant Phenotyping Experiments (MIAPPE) [20] is an community driven effort to agree on minimal information to describe plant phenotyping experiments to enable interoperability and re-use. The Breeding API (BrAPI) makes use of MIAPPE concepts and enrich them by genomics concepts to specify RESTful APIs. It aims to cover the majority of data domains for breeding applications in a FAIR manner. For crop breeding applications, BrAPI provides a standard interface for phenotype and genotype databases. This community driven project works continuously on a comprehensive API-specification by incorporating regularly feedback (https://brapi.docs.apiary.io) from data providers and data consumers. Since started in May 2014, BrAPI project is currently in active developing state and most recent stable release is version v1.3. All previous versions are also archived and can be accessed at https://github.com/plantbreeding/API. There are numerous changes has been made in v1.3 in comparison to previous versions. This includes for example minor format changes like “datatypes” is converted to “dataTypes”. Similarly, Get call on endpoint “Germplasm-search” is deprecated to “Germplasm”. Some of these deprecated endpoints and attributes are listed in Table 3. BrAPI is useful in many cases and can provide great ease when handling plant breeding data. There are many useful cases including field phenotyping apps, FAIR data portals, Data integration and exchange, etc.

**Table 3:** Examples for the evolution of API for FAIR-IPK implemented endpoints.

endpoint	V1.2	V1.3
GET /calls	datatypes	dataTypes
GET /breedingmethods	name	breedingMethodName
GET /germplasm-search	germplasm-search	germplasm
	genus	germplasmGenus
	species	germplasmSpecies
GET /germplasm/{germplasmDbId}/pedigree	parent1DbId	parent1Id
	parent2DbId	parent2Id

The BrAPI specification has a release cycle of 6 month. Here we show the changed attributes from version 1.2 to version 1.3 only. For instance, germplasm-search is replaced with germplasm, without affecting the data. There are also changes for endpoints itself, which is not documented in this example. As illustrated here, it can be assumed that there is a notifiable effort for BrAPI-endpoint provider to provide up-to-date endpoint implementations.

### 2.4 FAIR-IPK

**Figure 3:**
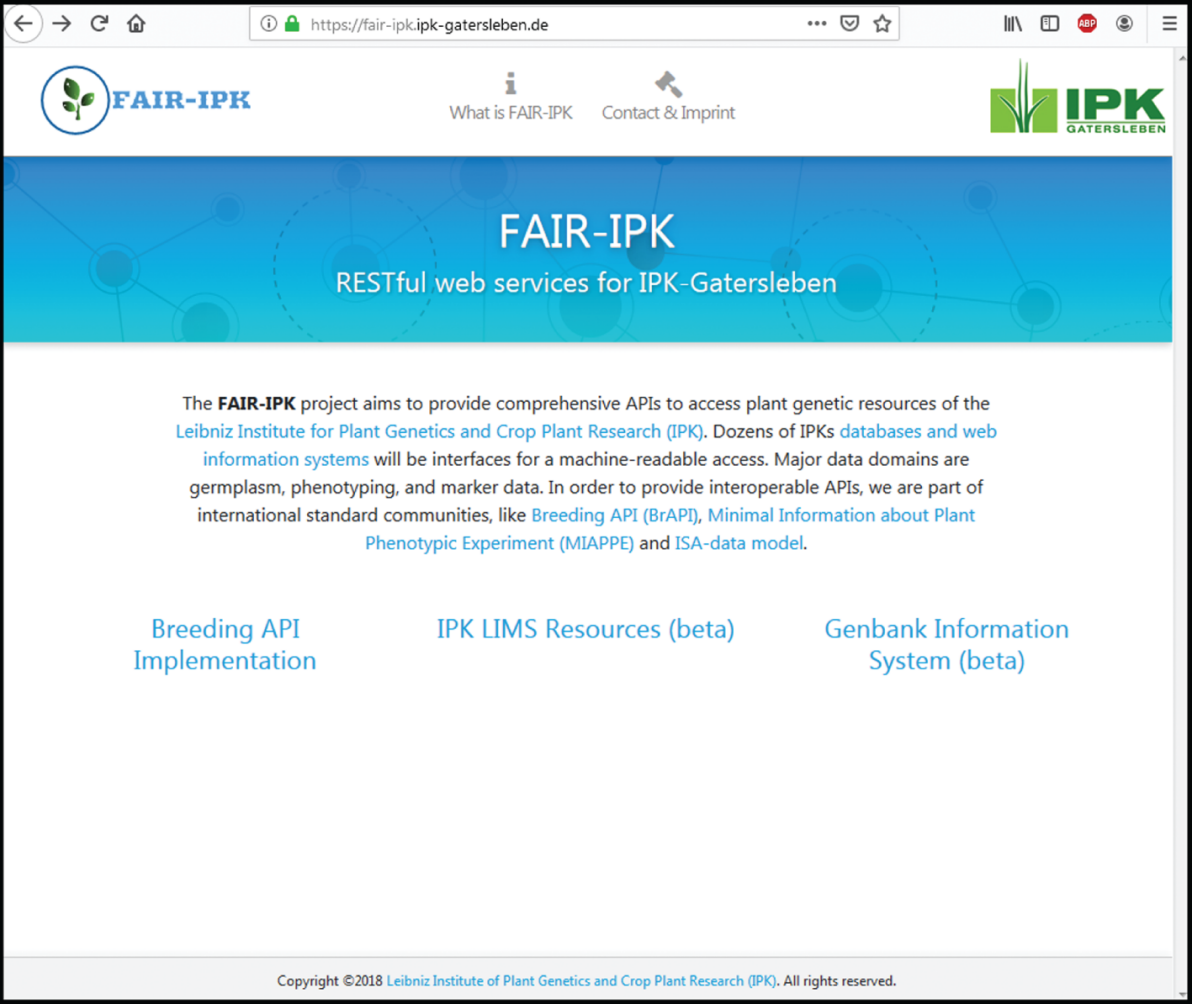
Web interface for accessing digital PGR of IPK-Gatersleben.

FAIR-IPK, (https://fair-ipk.ipk-gatersleben.de) was a core financed 12 person month project in 2018 to enable machine-readable access to IPK LIMS in a FAIR manner and show the benefits for the institute’s strategie towards an digital resource center. The project demonstrator use-case is the machine-readable access to plant phenotyping data, stored in IPK LIMS. All developed RESTful APIs are documented at the project web-site (see in Figure 3).

**Figure 4:**
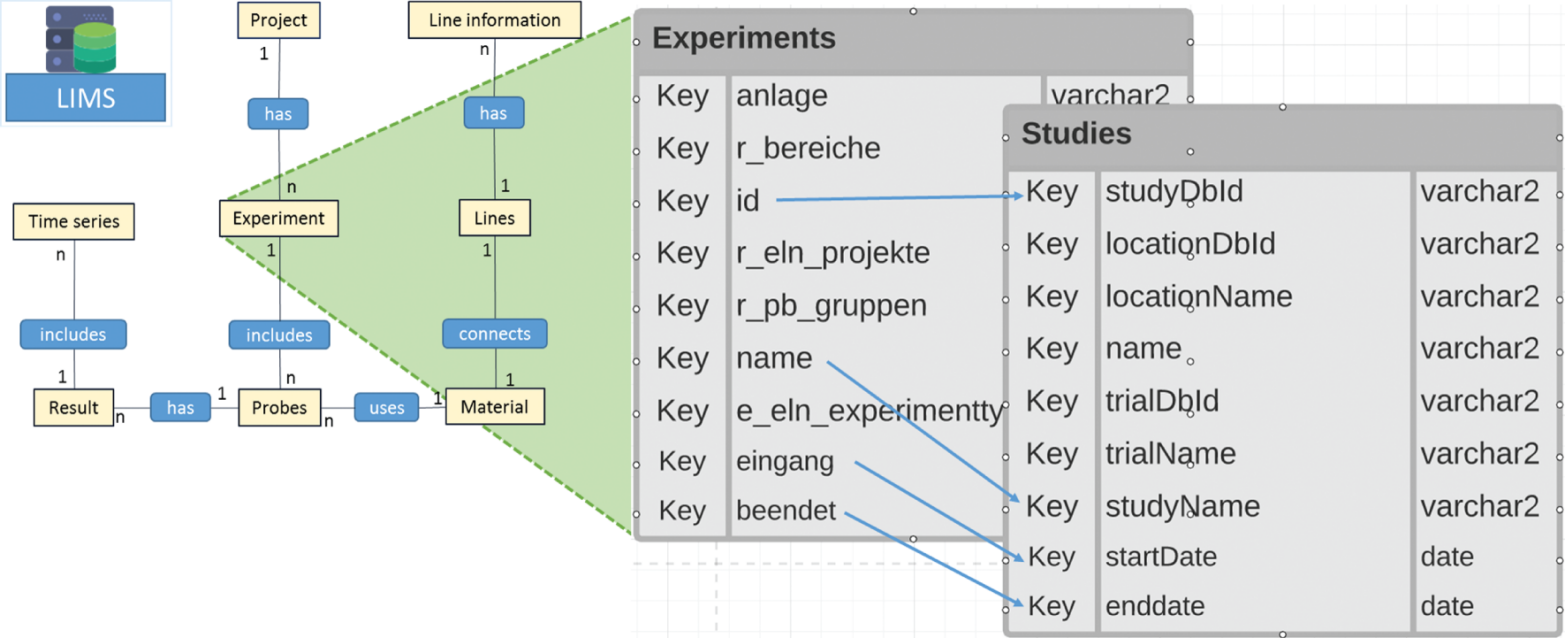
Mapping of LIMS resources to BrAPI resources.

**Figure 5:**
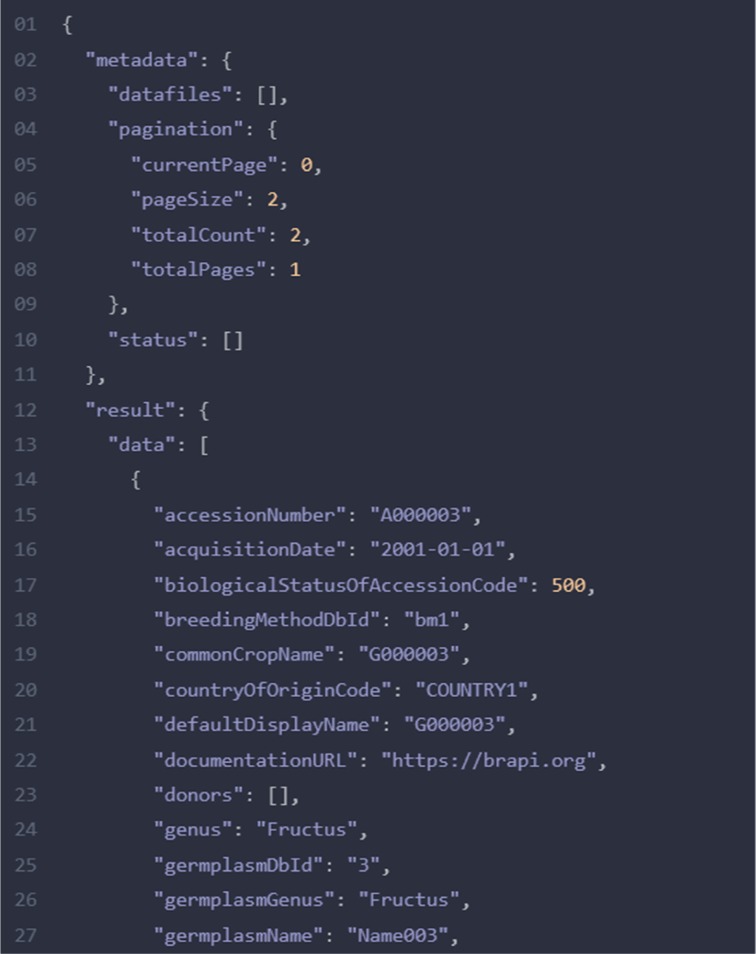
Example JSON output for the BrAPI call GET /germplasm-search – the first element comprise meta data, like status, linked data files and pagination information. The result part comprises the array of query results for that particular page.

SQL queries has been programmed to map LIMS data structure into unified SQL-views. As illustrated in Figure 4, a table “Experiments” from generic LIMS ER Schema mapped to BrAPI domain object “studies”. The arrow shows the corresponding possible mapping of attributes. The BrAPI-fied json output of such an entity is shown in Figure 5. This shows some of the attributes from BrAPI-complaint Germplasm resource.

## 3 Implementation using Model-View-Control paradigm

The FAIR-IPK project has been implemented using Grails framework, an industry approved, scalable web application development framework for platform independent, JAVA Virtual Machine (JVM) based runtime environments (https://grails.org/). It implements the Model-View-Controller (MVC) architecture and separates a web application, like as a RESTful service, strictly in three separate layers. The model layer is where data binding and back-end access resides, like the domain classes “Projects” and “Experiments” in the context of this paper. The view layer is the component that render data to the end user. A RESTful service usually render its output as JSON format. And finally, the controller, where all business logics are implemented, i.e. the communication with domain models, data transformation, aggregation, and validation.

**Figure 6:**
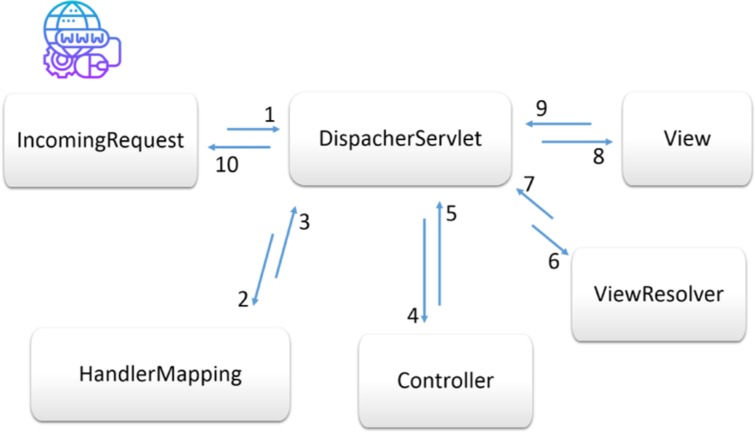
Flow of RESTful call in MVC-framework.

Adapted from Mohmmad Amr, Grails MVC Tutorial, 2014 (https://javabeat.net/grails-mvc-tutorial/).

The Grails frameworks principle of convention over configuration enables a well maintainable, expandable and sustainable RESTful services. Particularly, the Grails Object Relational Mapping (GORM) allows to avoid manual SQL programming for data queries and transformation into data objects. The use of plugins as dependencies in this framework enables developer to render the output data in different formats such as XML/JSON. The MVC framework concept is illustrated in Figure 6. Finally, Groovy, as Grails favourite JVM-code generating programming language, features with its simplified syntax, save navigation operator, automatic type conversion, etc.

Figure 6 (1) illustrate the workflow of RESTful data retrieval calls. In step (1) the request is redirected to the “DispatcherServlet” that acts as the controller for the “View”, “ViewResolver”, “Controller”, and “HandlerMapping” components. In step 2 and 3 request is delegated to the HandlerMapping that checks whether the requested call is valid and implemented. Next, the call is processed in the step 4 and 5 by the business logic that is implemented in the Controller. This includes for example, database queries, retrieval of domain class objects values and its mapping to predefined set of variables. In step 6 and 7 these variables along with their database mapped values are then bound the according view entities. The View Resolver is going to render the view entities in steps 8 and 9 is finally sent in step 10 to the client that initiated the RESTful service call.

### 3.1 Discussion

The transformation of over decades grown data management infrastructures into a digital resources center that features FAIR and machine readable data access is a process. In its present state, we demonstrated its benefit and elaborated in pilot studies required concepts, best practices and resources. As use case the phenotype data at IPK is already in transition towards fully FAIR and machine readable access using RESTful services [21]. To analyze and share plant phenotyping data, we utilizing BrAPI as a standard representation and API as robust and community accepted infrastructure. The clear advantage is to have a data-backend independence and avoid laborious, redundant and error prone implementation for every client application that requires access to IPK’s digital plant genetic and phenomics data resources. RESTful services are well integrated into different software development stacks. The implemented BrAPI endpoints enables a specific, pointwise as well as interoperable access to IPK’s LIMS managed database without proprietary data exports.

**Figure 7:**
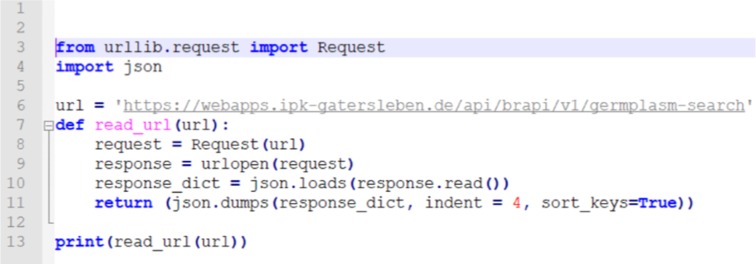
A Python Code Snippet to access RESTful endpoint Germplasm-search.

The Python (https://www.python.org/) code as shown in Figure 7 illustrates the direct access to the RESTful BrAPI endpoint “Germplasm-search”. The used libraries enables a seamless API call, data transfer, parsing of JSON formated result, transformation into Phyton objects. The required software libraries are available for all major programming languages. For the Phyton example, we used "urllib" and "json". The method "read_url" is parametrized with an URL, the JSON result is parsed and stored in the variable "response_dict" and finally output is rendered in a better readable format.

**Figure 8:**
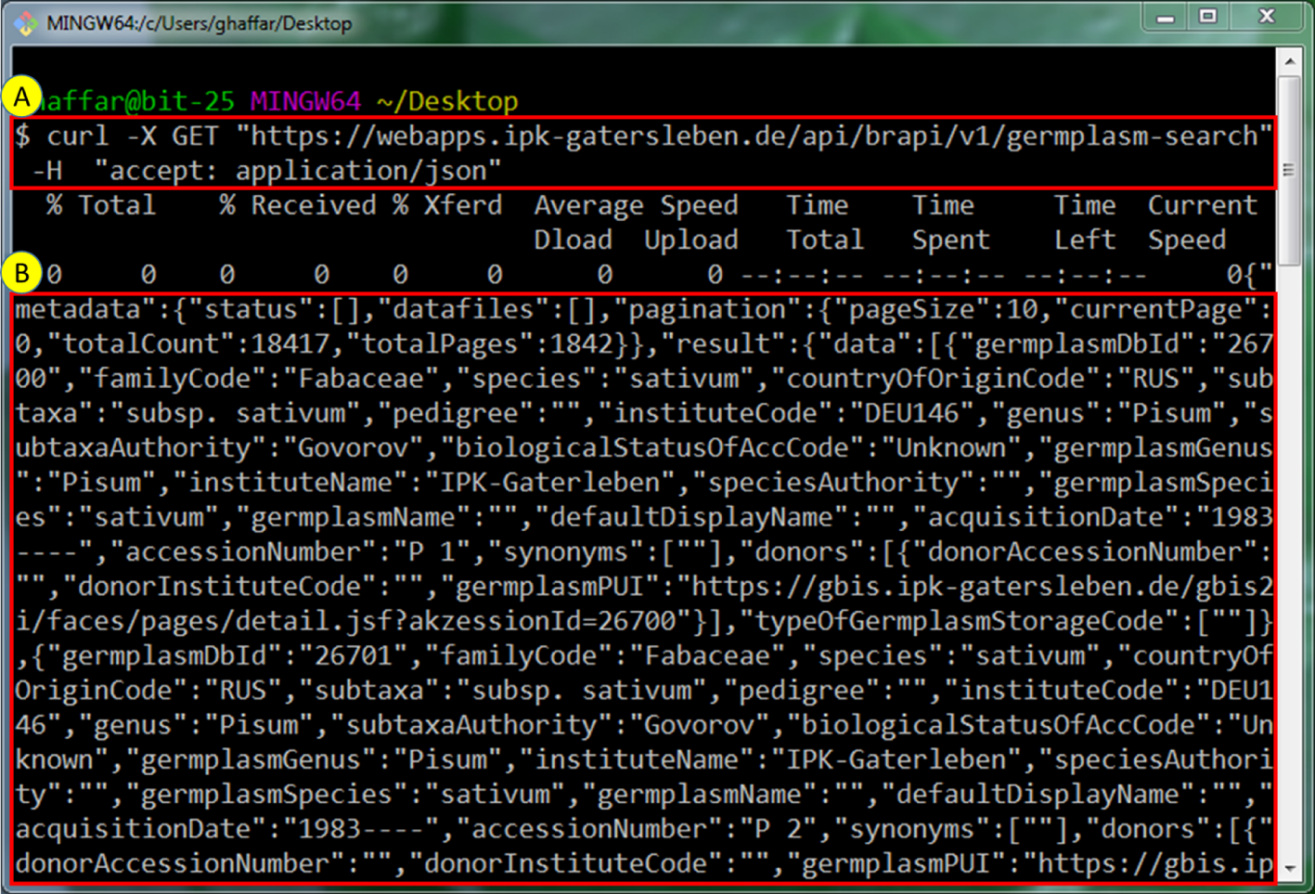
Accessing Germplasm resource REST endpoint using Linux cURL command line tool.

The REST endpoints can also be accessible through Linux command line interface tools like cURL (https://curl.haxx.se/). It is basically utilizing HTTP/HTTPS protocols and enables and ad-hoc REST endpoint testing or even Linux script based data processing. Figure 8A shows the actual cURL command to get access to the Germplasm-search REST endpoint. “X” is request method which is in this case “GET” request following the URL along with “H” header information. The header would accept different output formats which is in this case JSON format. Figure 8B shows the end result of the call that can retrieve JSON object when a GET request to that particular REST endpoint is sent.

### 3.2 Benchmark

**Figure 9:**
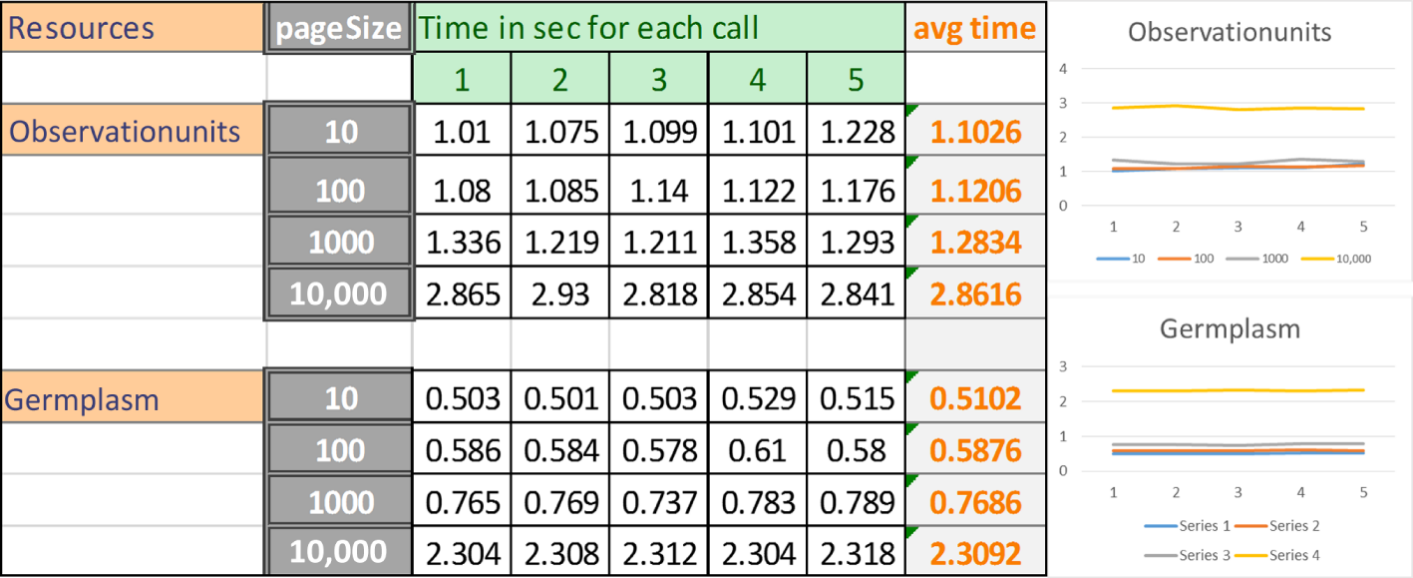
Performance test on large data-sets such as germplasm and observation units.

A benchmark has been performed to check the performance of some BrAPI calls implemented in this context. The test is initially focused on large data-set such as “observationunits” and “germplasm” resources. The remaining types of BrAPI call do not need to be tested as they are less complex and need less memory to charge data. Figure 9 shows these resources against their loading time in the memory in seconds. Each call is being called five times with increasing number of pageSize. PageSize refers to the number of samples or data-sets to be displayed on the screen or to end user. The average time is then calculated from these five calls per resources per pageSize. As the data-set size increases, the time complexity also increases. However, up to 1000 samples, the minimal average time of approximately one second is fair enough and consider to be fast and efficient.
